# ReFerm^®^: a postbiotic fermented oat gruel composition is reducing mast cell degranulation in the colon of patients with irritable bowel syndrome

**DOI:** 10.3389/fmed.2024.1408623

**Published:** 2024-07-04

**Authors:** Olga Biskou, Susanna Walter, Hans Israelsen, Martin E. Winberg, Olga Bednarska, Åsa V. Keita

**Affiliations:** ^1^Department of Biomedical and Clinical Sciences, Linköping University, Linköping, Sweden; ^2^Department of Gastroenterology, Linköping University Hospital, Linköping, Sweden; ^3^Department of Health, Medicine, and Caring Sciences, Linköping University, Linköping, Sweden; ^4^Nordic Rebalance A/S, Hillerød, Denmark

**Keywords:** functional gastrointestinal disorder, postbiotics, intestinal permeability, mucosal immunology, enteric nervous system

## Abstract

**Background:**

Irritable bowel syndrome (IBS) is a highly prevalent gastrointestinal disorder that affects ~4% of the global population. ReFerm^®^ is a postbiotic product derived from oat gruel fermented with *Lactobacillus plantarum 299v*, and it has been shown to have beneficial effects on intestinal permeability in patients with IBS. In this study, we investigated the effects of ReFerm^®^ on regulators of intestinal permeability, namely mast cells and enteric glial cells.

**Materials and methods:**

A total of 30 patients with moderate to severe IBS were treated with an enema containing ReFerm^®^ or a placebo twice daily. The patients underwent sigmoidoscopy with biopsies obtained from the distal colon at baseline and after 14 days of treatment. These biopsies were processed in two ways: some were fixed, embedded in paraffin, sectioned, and stained for mast cells and enteric glial cells; others were cryopreserved, lysed, and subjected to Western blotting to analyze the same markers.

**Results:**

Treatment with ReFerm^®^, but not the placebo, significantly reduced mast cell tryptase protein levels in the biopsy lysates. Although the number of mast cells remained unchanged in colonic biopsies, ReFerm^®^ treatment significantly reduced mast cell degranulation, a result not observed in the placebo group. Neither ReFerm^®^ or placebo treatment had an impact on total protein levels or the number of enteric glial cells in the biopsies.

**Conclusion:**

ReFerm^®^ treatment significantly reduced both total mast cell tryptase levels and the degranulation of mast cells in colonic biopsies from patients with IBS, suggesting a decrease in mast cell activity as a potential mechanism underlying the beneficial effects of ReFerm^®^. However, further research is required to assess the molecular mechanisms through which ReFerm^®^ operates in the colons of patients with IBS.

**Clinical trial registration:**

https://clinicaltrials.gov, identifier: NCT05475314.

## Introduction

Irritable bowel syndrome (IBS) is a functional gastrointestinal disorder characterized by abdominal pain and disturbed bowel habits, often combined with psychological symptoms like anxiety and depression (1). According to the Rome IV criteria (2), the prevalence of IBS is 4% globally, classifying it as one of the most common gut-brain axis disorders (3). IBS can be subdivided into four subgroups based on the predominant stool form: IBS with constipation (IBS-C), IBS with diarrhea (IBS-D), IBS with mixed bowel habits (IBS-M), and unclassified IBS (IBS-U) (3). Over time, IBS can progress from one subgroup to another (4). Although not life threatening, IBS causes a significant individual and socioeconomic burden (5–8) due to the lack of effective treatment. The main reason for the lack of effective treatment is the poor understanding of the pathophysiology of IBS. Contributing factors for IBS include microbial dysbiosis, increased intestinal permeability, and intestinal immune activation (9, 10).

Intestinal permeability is highly regulated, and some of the regulatory mechanisms lie within the lamina propria. Mast cells are known to play an important role in inflammatory bowel disease (11, 12) and are involved in visceral hypersensitivity in patients with IBS (13). Furthermore, mast cells are known to regulate intestinal permeability (14, 15). Several studies have implicated significantly increased mucosal mast cell counts in IBS patients compared to healthy controls. However, there are also some studies reporting no differences (16). We previously reported higher levels of mast cell tryptase (MCT) and an increased density of mast cells in patients with IBS compared to healthy individuals (17). In addition, we found that the translocation of live bacteria through the colonic mucosa is partly controlled by mast cells (17). It has become evident that enteric glial cells are involved in regulating intestinal permeability in humans (18). In our previous study, we have demonstrated increased intestinal permeability in colonic biopsies from healthy individuals by the enteric glial cell mediators (19). Further, we demonstrated an imbalance in the enteric glial cell-mast cell communication in patients with IBS, which might contribute to the pathophysiology of the disease (19). Interestingly, both mast cells and the enteric glial cells can sense their environment, including sensing microbial factors and food components, and respond to them by releasing mediators (18, 20).

It is becoming increasingly apparent that intestinal permeability is linked to diet (21). Although specific food groups are difficult to identify with the currently available methods, there is a growing body of evidence suggesting that probiotics (21) and postbiotics (22) have a beneficial effect on intestinal homeostasis. Probiotics include live bacteria, while postbiotics are dead cells and/or products of bacterial metabolism beneficial to humans, such as short-chain fatty acids (SCFAs). One of the well-studied bacterial species, *Lactobacillus plantarum*, is commonly found in dairy products, meat, and vegetables and is often used in food fermentation, like the production of cheese, fermented vegetables, and beverages (23). Additionally, *L. plantarum* has been shown to have promising results in improving IBS symptoms (24–26). The *L. plantarum 299v* strain has proven beneficial for human health and is used as a probiotic supplement for treating gastrointestinal disorders (23). It has also proven to be beneficial in IBS treatment (23, 27) and has been reported to promote the production of tight junction proteins and improve transepithelial electrical resistance in *in vitro* models (28, 29). In terms of postbiotics, *L. plantarum 299v* has been reported to produce lactic acid, acetic acid, and propionic acid (30). *L. plantarum 299v* is used to ferment an oat composition to manufacture ReFerm^®^ (also called Profermin^®^). ReFerm^®^ is a postbiotic food product rich in SCFA and other microbial metabolites.

We recently showed that ReFerm^®^, given as an enema for 14 days, reduced intestinal permeability in colonic biopsies from patients with IBS (31). In the present study, we aimed to investigate the underlying mechanisms that might be responsible for the improvement of the intestinal barrier by investigating this in the same patient cohort as in the previous study. We investigated the effect of ReFerm^®^ and placebo on the expressions of MCT and the enteric glial cell-associated proteins, glial fibril acid protein (GFAP), and calcium-binding protein β (S100β) using immunofluorescence and Western blotting.

## Materials and methods

### Study design

**Figure 1:**
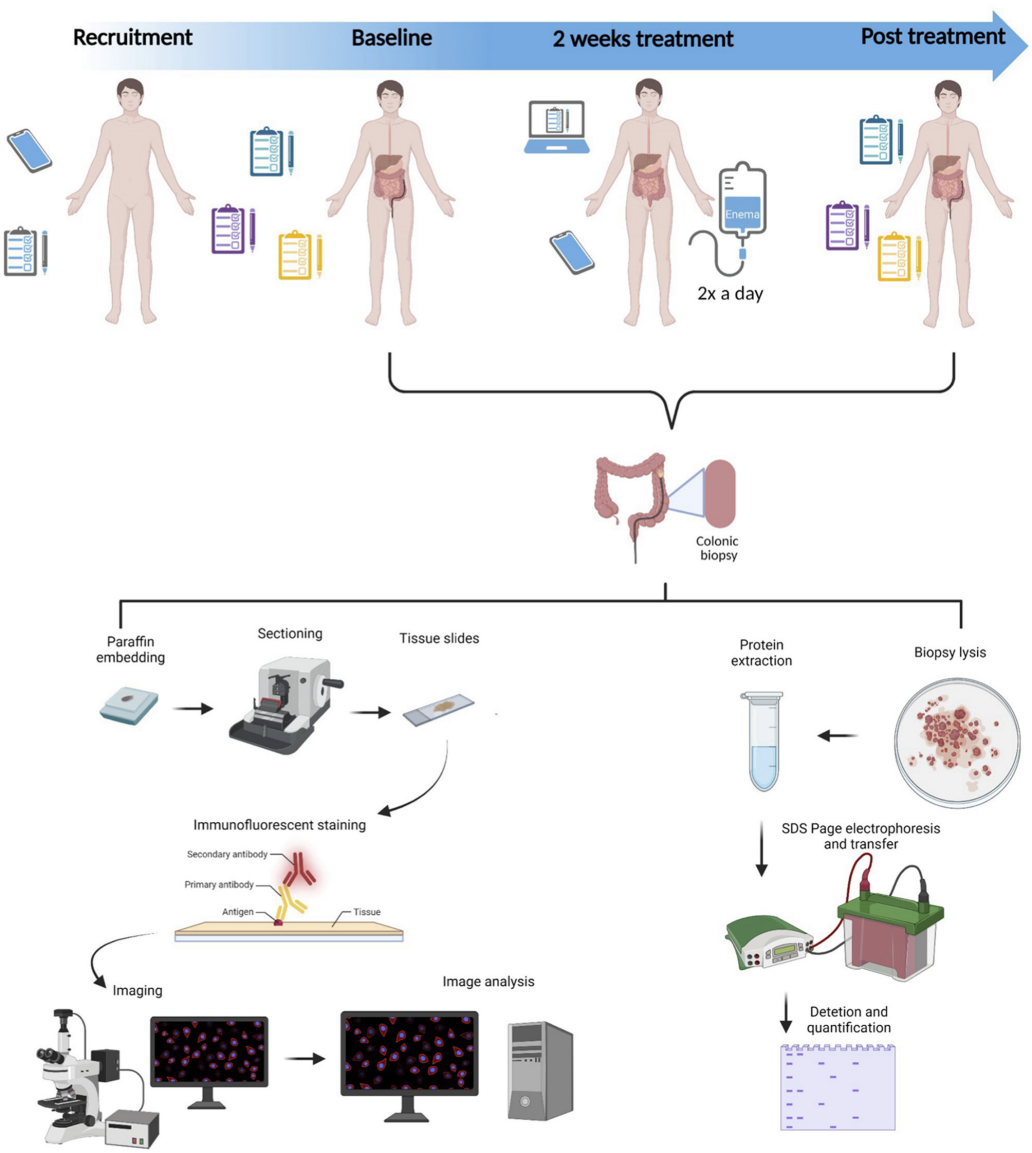
Study design. Patients with moderate-to-severe irritable bowel syndrome were recruited based on inclusion and exclusion criteria. The patients underwent two sigmoidoscopies that were 2 weeks apart, and in the meantime, they were administered enemas with either ReFerm^®^ or placebo twice a day for 2 weeks. The collected biopsies were either fixed, embedded in paraffin, sectioned, and stained with markers for mast cells and enteric glial cells or lysed and analyzed by western blotting for mast cells and enteric glial cell protein markers.

A single-blinded, randomized experimental study was conducted according to Figure 1. Patients with IBS were included based on the inclusion and exclusion criteria, as described in Table 1. As the patients acted as their own controls, self-reported allergy was not considered an exclusion criterion as long as both interventions were carried out with no allergic exposure. Patients were randomly allocated to either treatment with ReFerm^®^ or treatment with the placebo consisting of thickened water (see below for details). The patients who were included underwent sigmoidoscopy with biopsies collected from the distal colon at baseline and after 14 days of intervention with ReFerm^®^ or the placebo enema twice daily. The enema was administered rectally, in the left side body position, and retained at least 10 min, both in the left-sided position and when the patient was lying on the back with the face pointing upward, to activate retrograde peristalsis. Questionnaires were completed before and after the intervention to assess the clinical improvement of the symptoms. During the intervention, the patients completed daily questionnaires, as described in the questionnaire paragraph below. To improve compliance, patients received check-up calls from a principal investigator (OBe) twice per week during the intervention period. A consort flow diagram for the study can be found in Supplementary Figure 1.

**Table 1:** Inclusion and exclusion criteria for patients with IBS.

**Inclusion criteria**	**Exclusion criteria**
• Confirmed IBS-diarrhea or IBS-mixed bowel habits according to the Rome IV criteria. • Moderate to severe IBS according to the IBS-SSS score (≥175p) • Age 18–70 years • Fluency in written and spoken Swedish	• Organic gastrointestinal disease • Previous major gastrointestinal operations (apart from appendectomy and cholecystectomy) • Psychiatric disease (bipolar disease, schizophrenia) • NSAID intake < 2 weeks prior to the endoscopy • Self-reported pregnancy

IBS, irritable bowel syndrome; IBS-SSS, IBS severity scoring system; NSAID, non-steroidal anti-inflammatory drugs.

### Patients

A total of 30 patients (five men) meeting the Rome IV criteria (2), with a mean IBS duration of 13 years (range 2–40 years), were recruited, based on the inclusion criteria listed in Table 1, at the Gastroenterology Department, Linköping University Hospital, between December 2020 and April 2021. The mean age of the patients was 37 years (range 19–55 years), and the mean body mass index (BMI) was 26 (range 18–41). The patients were classified according to predominant bowel habits into IBS-D (*n* = 8) and IBS-M (*n* = 22) according to the Rome IV criteria. The mean symptom severity score (SSS) was 332.5 (range 180–488), classifying the patients as moderate to severe according to the IBS Symptom Severity Score (IBS-SSS) (32). The patients were randomly allocated to treatment with ReFerm^®^ (18 patients, of which two were men) or placebo (12 patients, of which three were men). There were no significant differences between the groups in terms of age, BMI, or disease severity. During the study, four patients (no men) dropped out of the ReFerm^®^ arm and two patients (one man) dropped out of the placebo arm. Consequently, the final number of patients was 14 (two men) in the ReFerm^®^ arm and 10 (two men) in the placebo arm. The study was approved by the Committee of Human Ethics (Dnr 2020-03485), and all participants provided their written informed consent.

### Sigmoidoscopy and biopsy collection

A flexible sigmoidoscopy was performed after routine preparation with an enema for bowel emptying. Sigmoidoscopy was performed with a scope inserted ~30–40 cm proximally from the *linea dentata*. Sigmoidoscopy was performed twice for each participant, at baseline and after 14 days of enema treatment with ReFerm^®^ or the placebo. Colonic biopsies were obtained with biopsy forceps without a central lance, directly placed in an ice-cold oxygenated Krebs buffer (115 mM NaCl, 1.25 mM CaCl_2_, 1.2 mM MgCl_2_, 2 mM KH_2_PO_4_, and 25 mM NaHCO_3_, pH 7.35), and immediately transported to the laboratory.

Biopsies from each patient were fixed immediately with 4% paraformaldehyde in PBS for 24 h at 4°C and subsequently maintained in 70% ethanol until they were embedded in paraffin and sectioned at 5 μm. In addition, biopsies were frozen at −80°C for Western blotting.

### The intervention product, ReFerm^®^, and the placebo product, Thick-it^®^

ReFerm^®^ was manufactured using a process consistent with previous methods (31, 33). In brief, the product would undergo a fermentation process and would then be tested for pH and colony-forming units (CFUs) of *Enterobacteriaceae*, yeasts/molds, and *L. plantarum 299v*. To pass the quality checks, the pH must be < 4.0, the CFU of *Enterobacteriaceae* and yeasts/molds must be < 100/ml, and the CFU for *L. plantarum 299v* must be >10^8^ immediately after the completion of fermentation. The energy content of ReFerm^®^ is 58 kcal (240 kJ) per 100 ml. The macronutrient content of ReFerm^®^ is 1.6 g of protein, 9.8 g of carbohydrate, and 0.9 g of fat per 100 ml. Each ReFerm^®^ package contains 250 ml.

To mimic ReFerm^®^, which possesses a larger viscosity than water, a commercially available product of thickened water was used as the placebo (Thick-it^®^ mildly thick, Kent Precision Foods Group, Inc.). Thick-it^®^ consists of artesian mineral water and ≤ 2% xanthan gum, calcium chloride, malic acid, potassium benzoate, potassium sorbate, sodium hexametaphosphate, and disodium ethylenediaminetetraacetic acid (EDTA). The energy content of one package of Thick-it^®^, 237 ml, is 5 kcal (21 kJ) from 0 g of protein, 1 g of carbohydrate, and 0 g of fat.

### Immunofluorescence

Slides with 5-μm tissue sections were incubated at 60°C for 2 h. For the staining of GFAP, S100β, and MCT, deparaffinization and rehydration were performed according to standard procedures. Antigen retrieval was performed by boiling for 20 min in citrate buffer (10 mM tri-sodium citrate dihydrate in H_2_O, pH 6, 0.5% Tween 20). After cooling down to room temperature, the sections were permeabilized using PBS supplemented with 0.1% Triton X for 10 min, followed by blocking with 1% BSA in PBS-0.5% Tween and 300 mM glycine for 30 min. Sections were individually stained with rabbit-anti-GFAP (1:500; Dako Cytomation, Glostrup, Denmark), rabbit-anti-S100β (1:200; Abcam, Cambridge, UK), or mouse-anti-MCT (1:200, Santa Cruz Biotechnology, USA) in 1% BSA in PBS-0.5% Tween and 300 mM glycine, overnight at 4°C. Following incubation, the primary antibody was washed away with PBS-0.5% Tween 20 and 300 mM glycine (wash buffer), and the sections were stained with Alexa Fluor 546-conjugated secondary antibodies (anti-mouse or anti-rabbit, 1:500; Invitrogen) for 1 h at room temperature, protected from light. To enable mast cell degranulation analysis, the cell membranes were stained using wheat germ agglutinin (WGA) Alexa Fluor 633 (Invitrogen, Stockholm, Sweden). WGA was incubated alongside the secondary antibody and diluted to a ratio of 1:500. The secondary antibodies were washed away, and the nuclei were stained using the NucBlue™ Fixed Cell Stain Ready Probe reagent (Invitrogen) for 10 min at room temperature, followed by washing and mounting using ProLong™ Glass (Invitrogen, Stockholm, Sweden). The slides were left to set at room temperature overnight, protected from light, and subsequently stored at 4°C.

### Imaging

Images, five fields of view per slide, were obtained for GFAP and S100β using Leica DMi8 (Leica, Wetzlar, Germany) with 63x magnification. Individual cells positive for GFAP or S100β were quantified in biopsies from 10 to 14 patients in each group.

Mast cells were quantified from confocal images with four fields of view per slide obtained from the Zeiss LSM 800 (Carl Zeiss, Jena, Germany) using 20x magnification. Individual mast cells, 10 per slide, were imaged from 7 to 8 patients who were randomly selected in each group (between 70 and 80 cells per group) using the Zeiss LSM 800 with 63x magnification and 4x digital zoom.

### Image analysis

The number of cells positive for GFAP, S100β, and MCT were quantified using CellProfiler (34), and the pipelines used can be provided upon request. The data are presented as the mean number of positive cells/fields of view.

The mast cell degranulation was assessed using Huygens Professional (Scientific Volume Imaging B.V., Netherlands). The images were deconvoluted using the deconvolution express option, and the deconvoluted images were analyzed using the object analyzer. The cell outline, WGA staining, was selected as the primary object, and the Gaussian filter was applied. The threshold and seed were only adapted if the outline of the cell was not clear. The MCT was selected as the secondary object. The segmentation was performed using the watershed filter. The seed was adjusted to 0, and the threshold was adapted to values between 5 and 8. Following the segmentation, the extracellular tryptase foci were selected manually, and their number and size were recorded. All imaged cells were included in the analysis. After removing the outliers, 52–66 mast cells were included in each condition. The individual rendering and the list of selected objects can be provided upon request.

### Western blotting

Total protein was extracted from frozen biopsies as previously described (35) using Radio-Immunoprecipitation Assay (RIPA) buffer (Thermo Fisher, Stockholm, Sweden). Denatured proteins, 20 μg per sample, were electrophoresed on 4–20% Tris-Glycine SDS-gel (Thermo Fisher, Stockholm, Sweden). Proteins were transferred to a nitrocellulose membrane (Amersham, Darmstadt, Germany) in Tris-Glycine buffer (Thermo Fisher, Stockholm, Sweden) supplemented with 20% ethanol. Following the transfer, membranes were blocked with a 5% blotting-grade blocker (Bio-Rad, Solna, Sweden) for 1 h at room temperature. Membranes were incubated overnight at 4°C with rabbit polyclonal antibody anti-GFAP (1:1,000, Dako Cytomation, Stockholm, Sweden), rabbit monoclonal anti-S100β antibody (1:1,000, Abcam, Cambridge, UK), mouse anti-mast-cell-tryptase (1:500, Santa Cruz Biotechnology, USA), mouse monoclonal anti-β-actin antibody (1:10,000; Cell Signaling, BioNordika, Solna, Sweden), in Tris-buffered saline (TBS) pH 7.6, 5% w/v BSA, and 0.05% v/v Tween 20. Membranes were washed and incubated with Alexa Fluor 790-conjugated donkey polyclonal-anti-rabbit (1:20,000, Thermo Fisher Scientific, Stockholm, Sweden) and Alexa Fluor 680-conjugated donkey polyclonal-anti-mouse secondary antibodies (1:20,000, Thermo Fisher Scientific, Stockholm, Sweden) for 1 h at room temperature in TBS pH 7.6, 5% w/v non-fat milk, and 0.05% v/v Tween 20. After washing, fluorescent bands were detected and quantified using Odyssey CLx and Image Studio software (LI-COR Biosciences, Lincoln, NE, USA). The levels of GFAP, S100β, MCT, and β-actin loading control were corrected to their brightest signal within each membrane, and all proteins were then normalized to β-actin corrected values. Values are given as the analyzed proteins MCT, GFAP, or S100β/β-actin.

### Statistical analysis

Comparisons were made using the GraphPad prism. Outliers were identified using the Remove outlier test (ROUT) method with the Q set at a 1% cutoff and were excluded from further analysis. The data presented have been checked for normality using the Kolmogorov–Smirnov test or the Shapiro–Wilkins test. Data are presented as the median and interquartile range (IQR), and comparisons between groups were performed using the Wilcoxon matched-pairs signed rank test.

## Results

### Intervention with ReFerm^®^ significantly reduced the total levels of MCT but not the number of mast cells in the colon of patients with IBS

**Figure 2:**
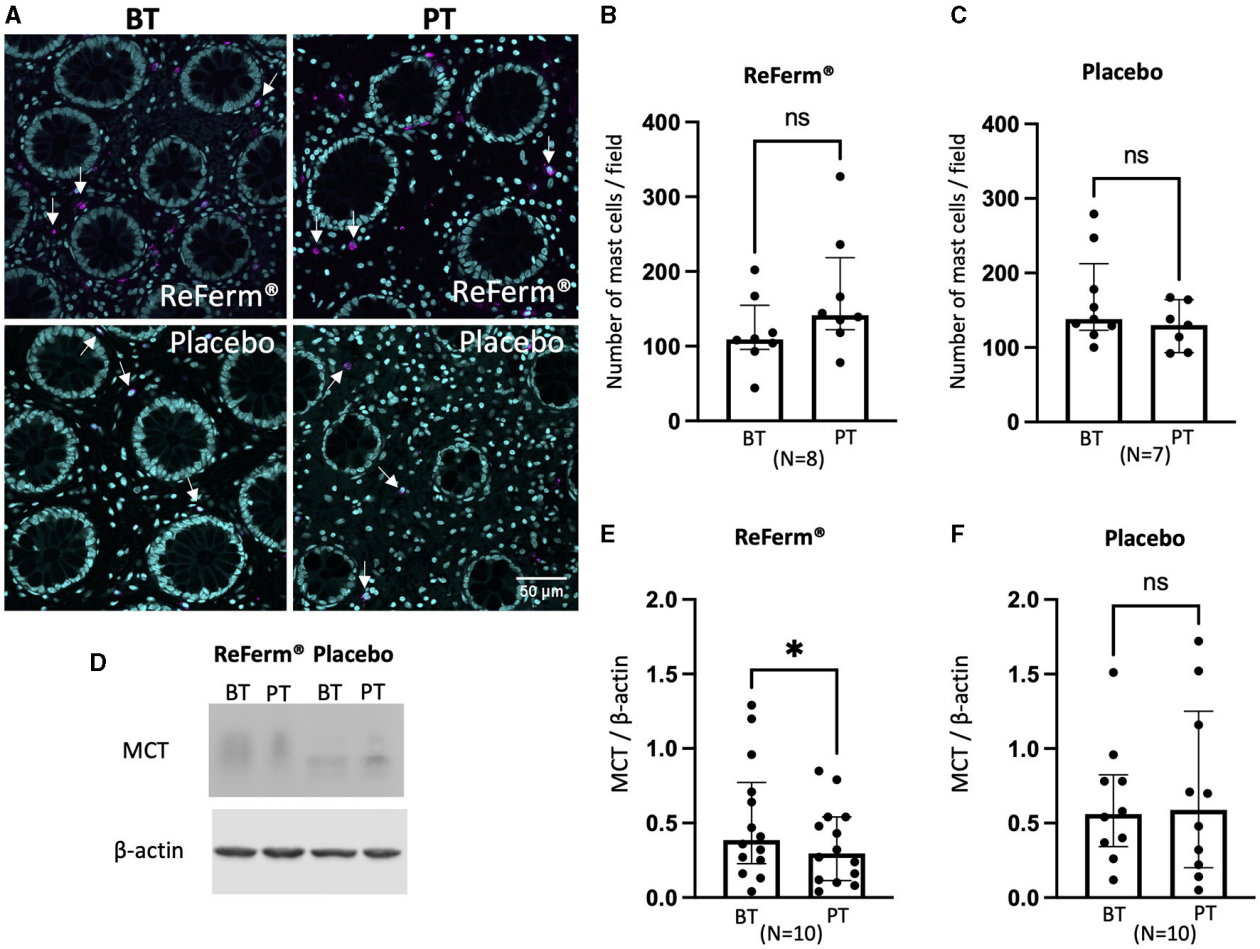
The effect of ReFerm^®^ and placebo on mast cells in patients with irritable bowel syndrome. **(A)** Representative fluorescence images of colonic biopsies stained for mast cell tryptase (MCT) before treatment (BT) and post treatment (PT). Magenta = MCT (arrows), cyan = cell nuclei stained by DAPI. **(B)** The number of mast cells was similar in biopsies BT and PT with ReFerm^®^. **(C)** The number of mast cells was similar in biopsies BT and PT with the placebo treatment. **(D)** Representative western blot membranes for MCT with β-actin as a loading control. **(E)** The quantification of western blotting for the ReFerm^®^ group showed significantly decreased MCT levels in PT compared to BT in the ReFerm^®^ group. **(F)** Quantification of western blots for the placebo group showed no differences between treatment groups. All data are given as a median and interquartile range, and comparisons are made using the Wilcoxon matched-pairs signed rank test. *p < 0.05. ns, non-significant; *N*, number of patients.

The first step was to investigate how mast cells were affected by ReFerm^®^ since mast cells are established regulators of intestinal permeability (15). Using MCT as a marker, we assessed the number of mast cells in the biopsies using immunofluorescence staining and image quantification (Figure 2A). The results showed that there was no difference in the number of mast cells at baseline and post-ReFerm^®^ treatment (Figure 2B), and the same applies to the placebo group (Figure 2C). Following immunofluorescence, the total MCT protein levels were assessed using Western blotting (Figure 2D). A reduction in MCT protein levels was observed post-ReFerm^®^ treatment, with a *p*-value of < 0.05 (Figure 2E), which was not observed in the placebo group (Figure 2F).

### Treatment with ReFerm^®^ resulted in a significant reduction in mast cell degranulation

**Figure 3:**
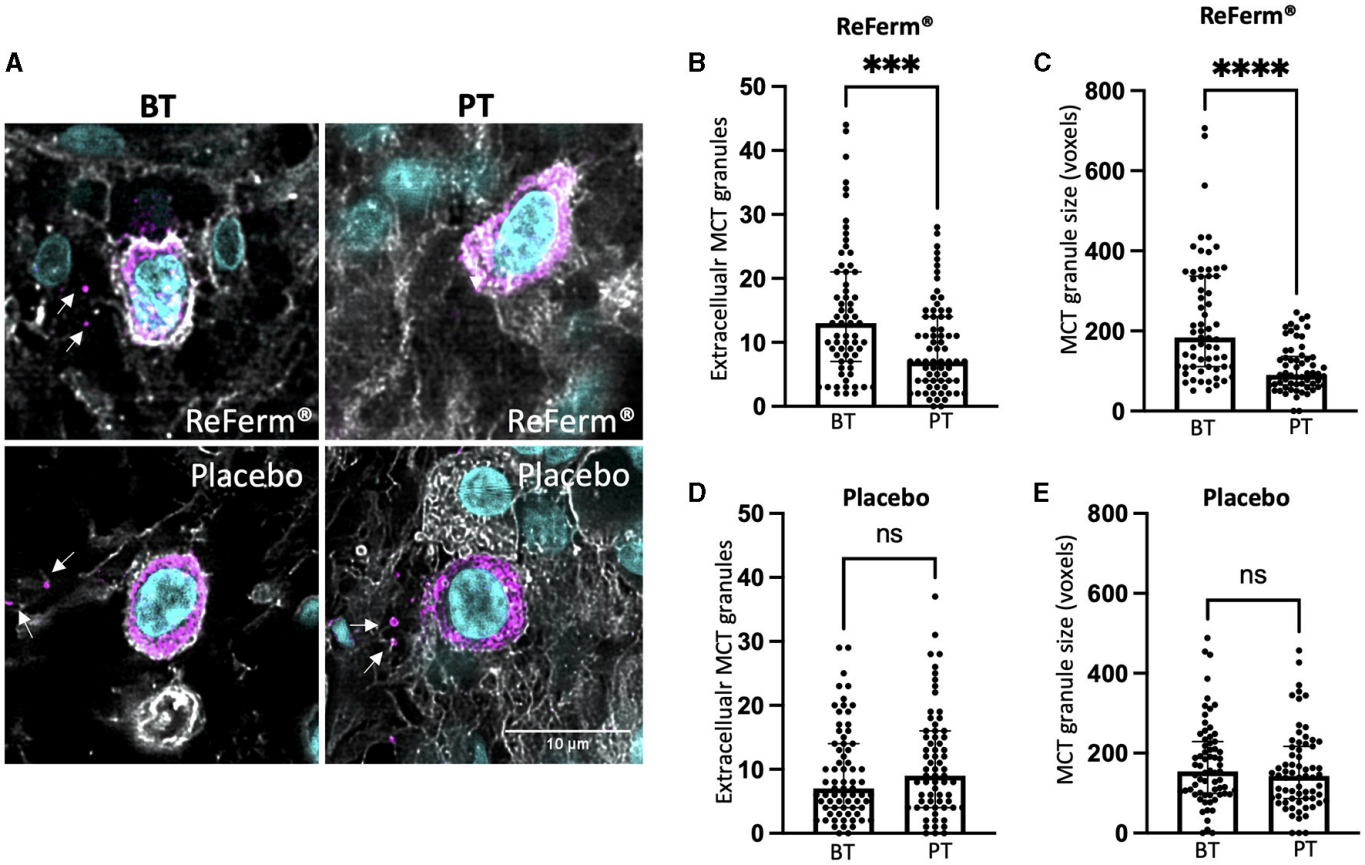
The effect of ReFerm^®^ and placebo on mast cell degranulation in patients with irritable bowel syndrome. **(A)** Representative images from single cells were double-stained for mast cell tryptase (MCT) and wheat germ agglutinin (WGA) to visualize the cell membrane. Magenta = MCT (arrows indicate extracellular granules), cyan = cell nuclei stained by DAPI. **(B)** The number of extracellular MCT granules was significantly decreased in the ReFerm^®^ group post treatment (PT) as compared to before treatment (BT). In total, 64 MCT+ cells were analyzed. **(C)** The size of MCT extracellular granules significantly decreased PT with ReFerm^®^ compared to BT. In total, 52 MCT+ cells were analyzed. **(D)** The number of extracellular MCT granules was not affected by the placebo treatment. In total, 62 MCT+ cells were analyzed. **(E)** The size of MCT extracellular granules was not affected by the placebo treatment. In total, 66 MCT+ cells were analyzed. All data are given as a median and interquartile range, and comparisons were made using the Wilcoxon matched-pairs signed rank test. ***p < 0.001, ****p < 0.0001, and ns, non-significant. Each point in the graphs represents one imaged and analyzed mast cell.

Since there were decreased total levels of MCT after ReFerm^®^ treatment but an equal number of mast cells, we intended to investigate whether the decreased MCT protein levels were associated with decreased mast cell activity. Individual double-stained cells for MCT and WGA were imaged and assessed for mast cell degranulation (Figure 3A). We observed a significant decrease, *p* < 0.001, in the number of extracellular MCT granules post-ReFerm^®^ treatment as compared to baseline (Figure 3B) and a significant reduction, *p* < 0.0001, in the size of the extracellular granules post-ReFerm^®^ treatment as compared to baseline (Figure 3C). There were no differences in the placebo group compared to baseline, neither for the number of extracellular MCT granules (Figure 3D) nor for the granule size (Figure 3E).

### The intervention with ReFerm^®^ did not affect the number of enteric glial cells in patients with IBS

**Figure 4:**
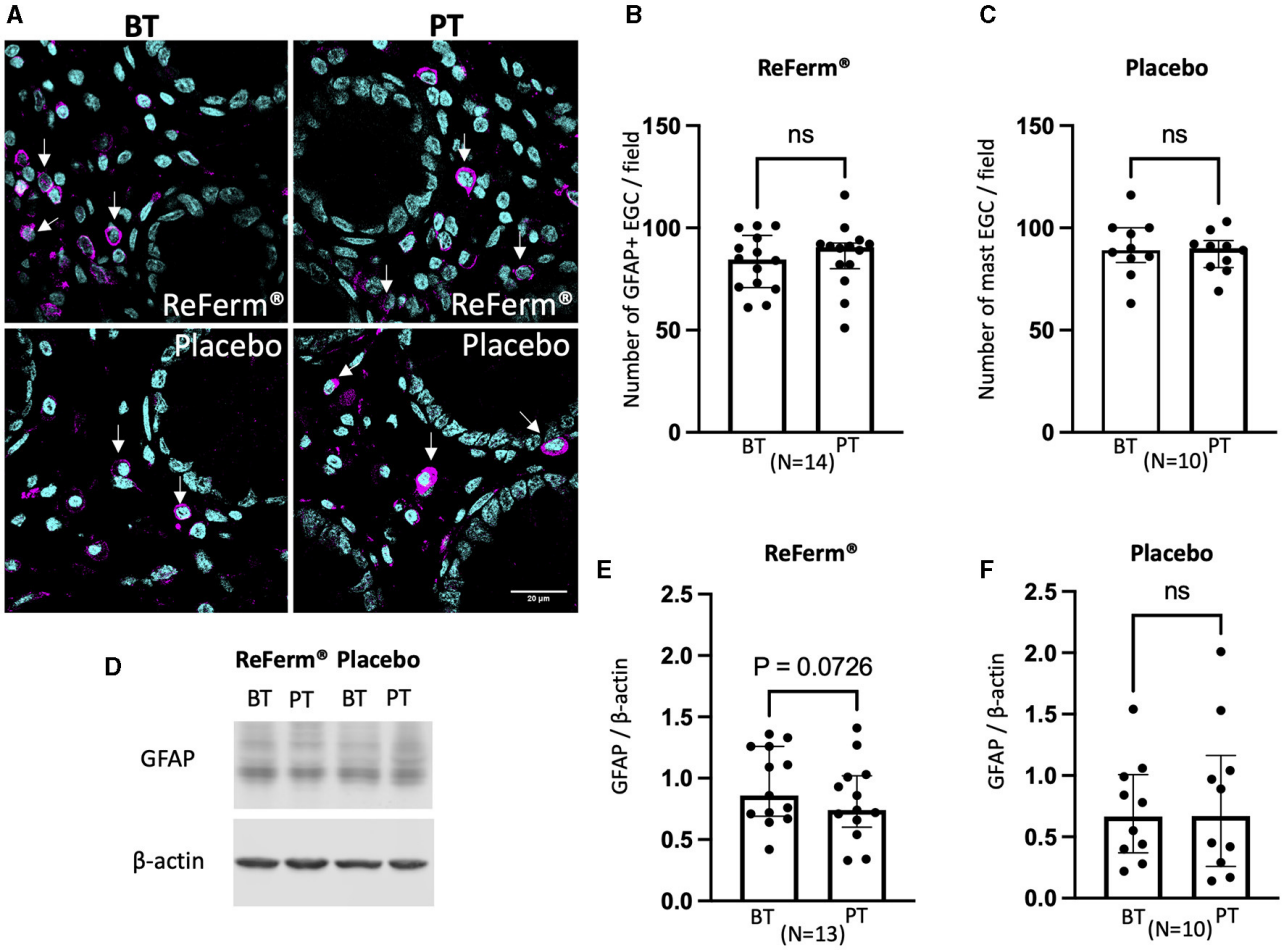
The effect of ReFerm^®^ and placebo on enteric glial cells identified by glial fibril acidic protein (GFAP) in patients with irritable bowel syndrome. **(A)** Representative fluorescence images of colonic biopsies stained for GFAP before treatment (BT) and post treatment (PT). Magenta = GFAP (arrows), cyan = cell nuclei stained by DAPI. **(B)** The number of GFAP^+^ cells was similar in biopsies BT and PT with ReFerm^®^. **(C)** The number of GFAP+ cells was similar in biopsies BT and PT with placebo **(D)** Representative western blot membranes for GFAP with β-actin as a loading control. **(E)** The quantification of western blotting for the ReFerm^®^ group showed decreased levels of GFAP PT compared to BT in the ReFerm^®^ group; however, it did not reach statistical significance. **(F)** The quantification of western blots for the placebo group showed no difference in GFAP levels in PT compared to BT. All data are given as a median and interquartile range, and comparisons are made using the Wilcoxon matched-pairs signed rank test. ns, non-significant; *N*, number of patients.

**Figure 5:**
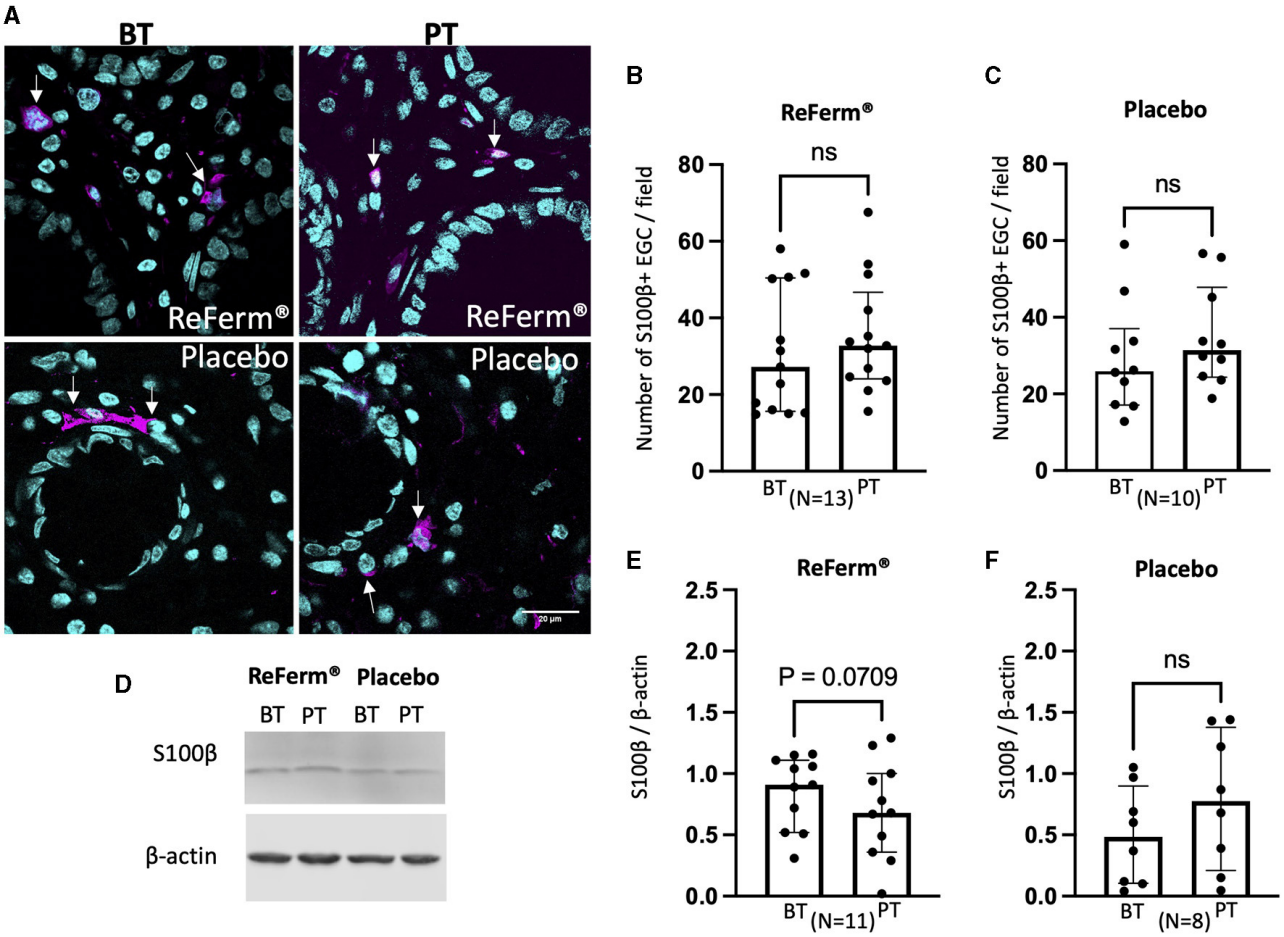
The effect of ReFerm^®^ and placebo on enteric glial cells identified by calcium-binding protein β (S100β) in patients with irritable bowel syndrome. **(A)** Representative fluorescence images of colonic biopsies stained for S100β before treatment (BT) and post treatment (PT). Pink = S100β (arrows), blue = cell nuclei stained by DAPI. **(B)** The number of S100β+ cells was similar in biopsies BT and PT with ReFerm^®^. **(C)** The number of S100β+ cells was similar in biopsies BT and PT with placebo **(D)** Representative western blot membranes for S100β+ with β-actin as a loading control. **(E)** Quantification of western blotting for the ReFerm^®^ group showed decreased levels of S100β PT compared to BT in the ReFerm^®^ group; however, it did not reach statistical significance. **(F)** Quantification of western blotting for the placebo group showed no difference in S100β levels in PT compared to BT. All data are given as a median and interquartile range, and comparisons are made using the Wilcoxon matched-pairs signed rank test. ns, non-significant; *N*, number of patients.

Subsequently, we investigated whether the intervention with ReFerm^®^ affected the number of enteric glial cells, another type of key regulator of intestinal permeability. Using GFAP and S100β as markers, we assessed the number of enteric glial cells in colonic biopsies using immunofluorescence staining and image quantification (Figures 4A, 5A). We did not observe changes in the number of GFAP^+^-enteric glial cells (Figures 4B, C) or S100β^+^-enteric glial cells (Figures 5B, C) in either of the treatment groups.

Western blotting results indicated that the amount of GFAP (Figures 4D–F) and S100β (Figures 5D–F) was not affected by the ReFerm^®^ or placebo treatment. However, there was a trend toward decreased levels of GFAP and S100β after ReFerm^®^ treatment (GFAP: *P* = 0.0726 and S100β: *P* = 0.0709), which could not be observed in the placebo group (GFAP: *P* = 0.6202 and S100β: *P* = 0.297) (Figures 4E, F, 5E, F).

## Discussion

In this study, for the first time, we showed that the postbiotic oat gruel fermented with *L. plantarum* 299v, ReFerm^®^, significantly reduced total MCT protein levels and the degranulation of mast cells in the colon of patients with moderate to severe IBS.

In our previous study (31), we showed that treatment with ReFerm^®^ decreased colonic paracellular permeability in patients with IBS and *in vitro* in Caco-2 cells. In the present study, we aimed to explain the mechanisms of ReFerm^®^ action in the colon of the same patients as included in our previous study by focusing on two cell types that are known as regulators of intestinal permeability, namely the mast cells (20) and the enteric glial cells (18).

Mast cells are involved in intestinal homeostasis as well as in gastrointestinal disease (15). Evidence in the literature indicates that the number of mast cells may be increased in the colon of IBS patients compared to healthy controls, but not necessarily in all regions of the colon (36). In the present study, we quantified the number of mast cells before and after ReFerm^®^ treatment. However, we did not observe differences in mast cell numbers. We previously showed increased mast cell numbers in the colon of patients with moderate to severe IBS (17, 37) and enhanced MCT levels in the biopsy lysates compared to healthy controls (17). As we have reported earlier in this study, ReFerm^®^ does not affect the number of mucosal mast cells in the sigmoideum of patients with IBS. However, the results showed a significant reduction of total MCT levels by Western blotting of biopsy lysates post-treatment with ReFerm^®^ but not with the placebo. Together, these data indicate that ReFerm^®^ affects the levels of MCT in the tissue without reducing the number of mast cells present. Stabilizing mast cells as well as targeting their mediators, such as tryptase, histamine, vasoactive intestinal polypeptide, or serotonin, has been of interest for many years (38, 39), demonstrating modestly promising results, corroborating the ground truth about the multifaceted etiology of the disease and the need for combination therapies. Our findings show decreased mast cell degranulation by ReFerm^®^, which further emphasizes the importance of mast cell activation in IBS pathophysiology.

When it comes to IBS, there is evidence corroborating the association between mast cell activity, rather than quantity, and IBS symptom severity (40). Accordingly, we assessed the degranulation status of mast cells at baseline and post-ReFerm^®^ treatment. We observed a significant reduction in the number and size of extracellular MCT granules post-treatment. A previous study summarized in a review (41) reported that dietary fibers and SCFA can modulate the activity of mast cells *in vitro*. Furthermore, Folkets et al. further investigated how SCFAs affect the activation of mast cells, and they reported that propionate and butyrate inhibit human mast cell activation *in vitro* (42). These observations may explain the reduction in mast cell degranulation observed in our study since ReFerm^®^ contains SCFAs, in particular, high amounts of lactic acid but low amounts of propionate and butyrate. However, lactic acid can easily be converted to butyrate in the intestine (43), which can subsequently provide beneficial effects of butyrate to patients.

Except for mast cells, enteric glial cells play multiple roles in the intestine, such as regulating the maintenance of motility, immune activation, and epithelial barrier integrity, and are involved in intestinal disease pathogenesis (18). However, little is known about enteric glial cell interactions with food components, the intestinal microbiome, prebiotics, probiotics, and postbiotics. In this study, we investigated whether ReFerm^®^ could affect the number of enteric glial cells or total levels of enteric glial cell markers GFAP and S100β in the colon of patients with IBS. A previous study from a type 2 diabetic mouse model showed that the GFAP levels in the colon, the amygdala, and the prefrontal cortex were significantly increased compared to healthy mice, and supplementation with an unknown strain of *L. plantarum* combined with inulin resulted in a significant decrease in GFAP levels in all three sites (44). In the present study, we did not study the effects of postbiotics on the brain. However, in the colon, we demonstrated that the human body responds differently compared to mice since ReFerm^®^ treatment did not result in the reduction of enteric glial cell numbers or reduced levels of enteric glial cell proteins using western blotting. A plausible reason for the diverse results obtained may be the fact that ReFerm^®^ is a postbiotic containing *L. plantarum* 299v metabolites produced by oat fermentation in contrast to a probiotic *Lactobacilli* combined with inulin. Additionally, the evidence in the literature about how enteric glial cells are affected by the gut microbiome (45), prebiotics, and probiotics (44) comes primarily from animal studies, and this body of evidence is not necessarily representative of what is occurring in the human intestine, as recently shown (46). Although not significant, ReFerm^®^ treatment reduced the protein levels of GFAP and S100β, a result not observed in the placebo group. This observation indicates that ReFerm^®^ might influence the enteric glial cells, but it did not reach statistical significance, possibly due to the limited number of patients.

In addition, we did not study whether ReFerm^®^ can modulate the function or type of the enteric glial cells present in the colon of patients with IBS. This question needs to be further investigated, but it might carry several challenges since it is not yet well-established how many different enteric glial cell subtypes are present in the human colon, and there is a lack of specific markers for the different enteric glial cell subtypes (47). ReFerm^®^ and the effects it has on the colonic mucosa, as presented in this study, result from oat fermentation products, which is in line with previously described theories by Mukherjee et al. (48). Oat fermentation products involve a combination of native oat parts, fermented oat parts, and microbial metabolites formed during fermentation. The placebo used in the present study is composed of thickened water and lacks bacterial components, metabolites, and nutritional components. When choosing a product that would be used as the placebo in this study, we aimed to find a product that was neutral to the colonic milieu and was not plain water, as the patients were informed that ReFerm^®^, although nameless at the time, was based on oats. We, therefore, selected the thickened water Thick-it^®^ as the placebo to minimize the components with potential effects on the colonic mucosa. Since the placebo did not affect the colonic epithelium, as we showed, we conclude that the effects we demonstrate in the ReFerm^®^ group were the result of the active components within ReFerm^®^.

There are several other potential confounding factors that could influence our results. Recent studies demonstrated that immune-mediated reactions such as mast cell and eosinophil activation might be triggered by certain foods (20). Vanuytsel et al. hypothesized that IBS is part of a spectrum of food-induced disorders mediated by mast cell activation (20). On the other hand, it is well-known that diet is the key regulator of the gut microbiome (21). Unfortunately, we did not collect any baseline dietary or microbiome data in our study. Moreover, several medications have the potential to influence mast cells, which we did not control for in our study, as we assumed that every participant serves as his/her own control, which eliminates the importance of the baseline data, such as diet, medication, or even lifestyle factors such as daily stress intensity, given the invariance of those parameters during the relatively short study period. In addition to the previously described limitations, one key limitation of this study is that we are only presenting observational data without investigating the underlying mechanism of ReFerm^®^ action. Studying the mechanisms resulting in the reduction of mast cell degranulation due to ReFerm^®^ requires further research using *in vitro* and *in vivo* models. Furthermore, this study is only focused on patients with IBS without investigating whether ReFerm^®^ has a similar beneficial effect in other gastrointestinal conditions.

We previously reported that ReFerm^®^ significantly improves the colonic barrier in biopsies from patients with IBS (31). In this study, using biopsies from the same patient cohort of patients, we observed a significant reduction in MCT protein levels and reduced mast cell degranulation, shedding light on the novel field of postbiotics and immunoregulation in IBS. Further studies are needed to assess how postbiotics like ReFerm^®^ modulate the intestinal microenvironment in gastrointestinal diseases and syndromes such as IBS and to determine whether this modulation translates into significant benefits for patients. In the present study, the patients were asked to use ReFerm^®^ via an enema, allowing us to directly study its effects on the human colonic mucosa. However, further studies are needed to assess the beneficial effects of ReFerm^®^ when administered orally to IBS patients over a period longer than 2 weeks to assess different methods of delivery and their therapeutic effects.

## Conclusion

In this study, we evaluated how a postbiotic fermented oat gruel composition called ReFerm^®^ affected the colon of patients with IBS. We assessed the effects of ReFerm^®^ on mast cells and enteric glial cells. ReFerm^®^ does not affect the numbers of enteric glial cells or mast cells. However, we report that the total MCT protein levels and mast cell degranulation in colonic biopsies are significantly decreased as a result of ReFerm^®^ treatment in patients with IBS. Further studies are required to elucidate if ReFerm^®^ has similar effects on the colon of patients with IBS when used as an oral supplement. Our findings, further emphasizing the importance of mast cells' activation in IBS, may pave the way for plausible future treatment of patients with IBS. However, more research is needed to provide a deeper understanding of the mechanisms involved in the beneficial effects of ReFerm^®^.

## Data availability statement

The raw data supporting the conclusions of this article will be made available by the authors, without undue reservation.

## Ethics statement

The studies involving humans were approved by Etikprövningsmyndigheten; Box: 2110 75002; Uppsala. Contact: registrator@etikprovning.se. Telephone: 0046 10-475 08 00. The studies were conducted in accordance with the local legislation and institutional requirements. The participants provided their written informed consent to participate in this study.

## Author contributions

OBi: Methodology, Visualization, Writing – original draft, Writing – review & editing, Data curation, Formal analysis, Software, Validation. SW: Funding acquisition, Investigation, Project administration, Writing – review & editing, Conceptualization, Resources. HI: Methodology, Writing – review & editing, Conceptualization, Funding acquisition, Project administration, Resources. MW: Writing – review & editing, Data curation, Formal analysis, Methodology. OBe: Funding acquisition, Methodology, Project administration, Writing – review & editing, Investigation, Visualization. ÅK: Methodology, Writing – review & editing, Conceptualization, Funding acquisition, Investigation, Project administration, Resources, Supervision, Visualization, Writing – original draft.
