# Supplementary material for: ReFerm®: a postbiotic fermented oat gruel composition is reducing mast cell degranulation in the colon of patients with irritable bowel syndrome
Source: Front Med (Lausanne). 2024 Jul 4;11:1408623. doi: 10.3389/fmed.2024.1408623 (PMC11255971; doi:10.3389/fmed.2024.1408623)

Study Flow Diagram

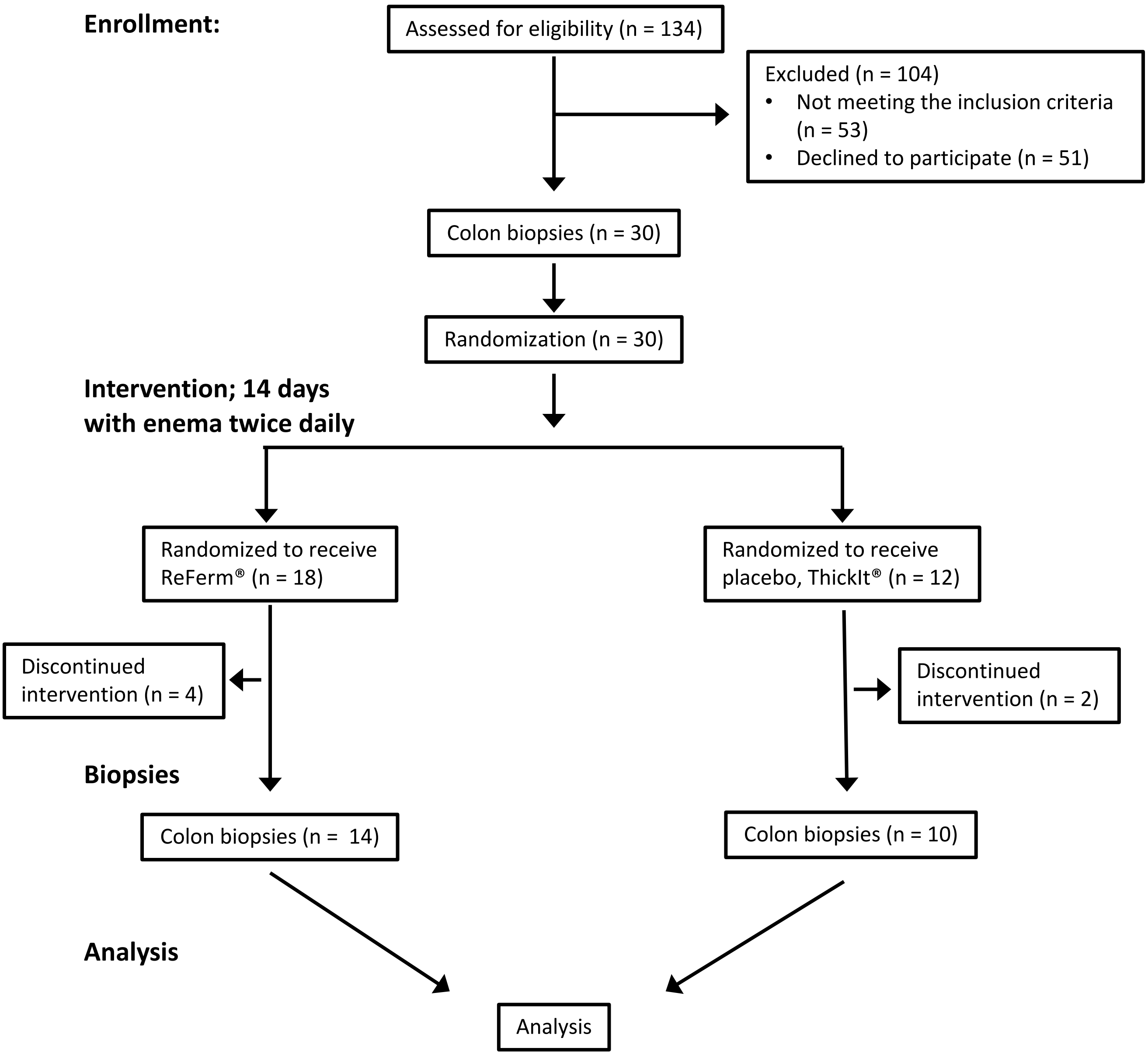

Supplement: Supplementary file 1 [file Data_Sheet_1.PDF]
